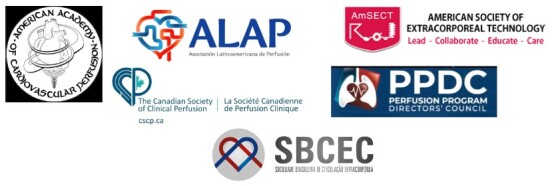# Normothermic regional perfusion and ex vivo perfusion position – endorsed

**DOI:** 10.1051/ject/2025044

**Published:** 2025-12-17

**Authors:** William D. Riley, Emily Saulitis Collins, Kirsten R. Kallies, Emily L. Thunstrom-Kahring, David Boyne, Jwana Ibsies, Scott M. Noesges, Caleb Varner, Phillip Bailey

**Affiliations:** 1 The American Board of Cardiovascular Perfusion Milwaukee WI 53202-3823 USA


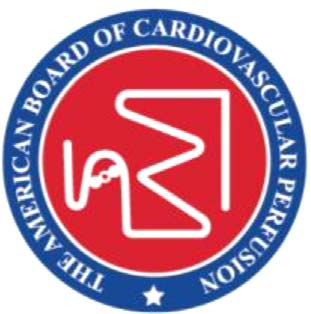


Dear Editor,

The American Board of Cardiovascular Perfusion released the following position statement in August 2024.

As a fundamental goal of our mission, the American Board of Cardiovascular Perfusion (ABCP) strives to develop and maintain quality standards in cardiovascular perfusion that promote the safety and protection of the public. These standards are attained through the enhancement of structured medical education, clinical rotations, and certification through an exam process to obtain the status of Certified Clinical Perfusionist (CCP). For nearly 50 years, these standards have been set and demonstrate the importance of structured education, similar to what other medical disciplines have shown as well.

CCPs hold a distinct and critical function in cardiac, thoracic, and other complex surgeries for both pediatric and adult populations. As Perfusionists, our practice is complex, involving mechanical knowledge of extracorporeal circuitry and medical sciences including anatomy, physiology, chemistry, cellular biology, pharmacology, and many other areas contributing to optimal end-organ perfusion. CCPs are uniquely versed in cardiopulmonary bypass (CPB) and extracorporeal membrane oxygenation (ECMO), as this is the basis and standard for cardiac surgical needs. As medical technology and science continue to push boundaries, the perfusion community has been called upon as caregivers in different arenas, including, but not limited to, chemotherapy perfusion and ECMO in the perioperative and even out-of-hospital setting by transporting patients via ground, rotor, or fixed-wing applications.

Most recently, CCPs have been at the epicenter of advances in organ procurement, including Normothermic Regional Perfusion (NRP) and ex vivo perfusion. NRP and ex vivo are areas perfectly suited for CCPs to utilize their education, experience, and other benefits of clinical certification to support patient safety and overall operative success. NRP and ex vivo perfusion are similar in all respects as defined by the basis of training and application of clinical knowledge. Ex vivo perfusion devices can be defined as a small CPB system, utilizing all the same processes and technology required for whole-body end-organ perfusion, but for a specific single organ, each with its own physiologic profile. As devices vary in organ flow and pressure regulation requirements, the CCP must respond in a manner very similar to patients undergoing CPB. NRP is an in vivo process to reanimate and preserve organs, and similar to ex vivo, this process includes the use of a modified or standard CPB machine and disposables. The NRP process is very complex and still developing in all regards to organ resuscitation, reanimation, and reperfusion.

The depth and breadth of understanding of end-organ perfusion, organ preservation, and successful management of CPB should not be taken lightly. The educational level and clinical expertise of the current generation of CCPs are at an all-time high and should be utilized in all processes of organ perfusion, whether in situ, ex vivo, or in vivo, for the benefit and safety of the public.

In summary, CCPs are the only medical professionals formally educated and trained in adult and pediatric extracorporeal technology and the many ancillary techniques and technologies applied in procedures requiring such support. Annual recertification through the ABCP maintains the high standards required to practice safely and effectively in these high acuity medical scenarios. The emerging technologies of ex vivo organ procurement and NRP have enhanced the viability of donated organs, and the trajectory of these techniques appears to be robust. As the only medical professionals with the appropriate training, clinical experience, continuing education, and professional support necessary to reliably and optimally perform these critical procedures, the American Board of Cardiovascular Perfusion insists on utilizing CCPs for these procedures.

Endorsed by: